# Adhesion of Annexin 7 Deficient Erythrocytes to Endothelial Cells

**DOI:** 10.1371/journal.pone.0056650

**Published:** 2013-02-20

**Authors:** Majed Abed, Siraskar Balasaheb, Syeda Tasneem Towhid, Christoph Daniel, Kerstin Amann, Florian Lang

**Affiliations:** 1 Department of Physiology, Eberhard-Karls-University, Tuebingen, Germany; 2 Department of Physiology, Medicine Faculty, Al-Furat University, Deir Ezzor, Syria; 3 Institute of Pathology, Friedrich-Alexander-University, Erlangen-Nuernberg, Germany; National Cancer Institute, United States of America

## Abstract

Annexin 7 deficiency has previously been shown to foster suicidal death of erythrocytes or eryptosis, which is triggered by increase of intracellular Ca^2+^ concentration ([Ca^2+^]_i_) and characterized by cell shrinkage and cell membrane scrambling with subsequent phosphatidylserine exposure at the cell surface. Eryptosis following increase of [Ca^2+^]_i_ by Ca^2+^ ionophore ionomycin, osmotic shock or energy depletion was more pronounced in erythrocytes from annexinA7-deficient mice (*anxA7^−/−^*) than in erythrocytes from wild type mice (*anxA7^+/+^*). As phosphatidylserine exposure is considered to mediate adhesion of erythrocytes to the vascular wall, the present study explored adhesion of erythrocytes from *anx7^−/−^* and *anx7^+/+^*-mice following increase of [Ca^2+^]_i_ by Ca^2+^ ionophore ionomycin (1 µM for 30 min), hyperosmotic shock (addition of 550 mM sucrose for 2 hours) or energy depletion (removal of glucose for 12 hours). Phosphatidylserine exposing erythrocytes were identified by annexin V binding, cell volume estimated from forward scatter in FACS analysis and adhesion to human umbilical vein endothelial cells (HUVEC) utilizing a flow chamber. As a result, ionomycin, sucrose addition and glucose removal all triggered phosphatidylserine-exposure, decreased forward scatter and enhanced adhesion of erythrocytes to human umbilical vein endothelial cells (HUVEC), effects significantly more pronounced in *anx7^−/−^* than in *anx7^+/+^*-erythrocytes. Following ischemia, morphological renal injury was significantly higher in *anx7^−/−^* than in *anx7^+/+^*-mice. The present observations demonstrate that enhanced eryptosis of annexin7 deficient cells is paralleled by increased adhesion of erythrocytes to the vascular wall, an effect, which may impact on microcirculation during ischemia.

## Introduction

Annexin A7 (or annexin VII, synexin), a member of Ca^2+^- and phospholipid-binding intracellular proteins [1]–[3] associates with secretory vesicles and serves as a Ca^2+^/GTP sensor in the regulation of secretion [4]–[6]. An initial attempt to create an annexin A7 knockout mouse yielded mice which were lethal at embryonic day 10, as anx7-deficient mutants died in utero from cerebral hemorrhage [7]. The heterozygous mice expressing only low levels of Anx7 protein were viable and fertile. The main molecular consequence of lower anx7 expression was a profound reduction in IP3 receptor expression and function in pancreatic islets [7]. Islet ß cell number and size were increased, presumably due to compensation of impaired insulin secretion by individual defective pancreatic ß cells [7]. In a further attempt, the homologous recombination of embryonic stem cells yielded a viable annexin A7 knockout mouse with mild phenotpye [8]. The phenotype included altered effects of heart rate on cardiac action potential [8] and enhanced glial cell proliferation [9]. Moreover, erythrocytes from annexin A7 knockout mice were shown to be more suseptible to eryptosis [10], [11], the suicidal erythrocyte death, which is characterized by cell shrinkage and cell membrane scrambling with exposure of phosphatidylserine at the cell surface [12].

Triggers of eryptosis include activation of Ca^2+^-permeable cation channels with subsequent increase of cytosolic Ca^2+^ concentration [13]–[21]. The cation channels are activated by PGE_2_ [22]. The increase of cytosolic Ca^2+^ activates Ca^2+^-sensitive K^+^ channels [23], [24] leading to exit of KCl with osmotically obliged water and thus to cell shrinkage [25]. Cytosolic Ca^2+^ further stimulates scrambling of the cell membrane with subsequent phosphatidylserine exposure at the cell surface [21], [26]–[28]. Cell membrane scrambling and thus phosphatidylserine exposure is influenced by several kinases, such as AMP activated kinase AMPK [17], cGMP-dependent protein kinase [29], Janus-activated kinase JAK3 [30], casein kinase [31], [32], p38 kinase [33], PAK2 kinase [34] as well as sorafenib [35] and sunifinib [36] sensitive kinases.

Phosphatidylerine exposing cells may adhere to endothelial cells [37] with resulting impairment of microcirculation [38]–[42] and, at least in theory, impact on reperfusion and tissue injury following transient ischemia. The present study thus explored, whether the enhanced susceptibility of annexin7 deficient erythrocytes to eryptosis is paralleled by enhanced adherence to human umbilical vein endothelial cells (HUVEC) and altered renal injury following ischemia.

## Materials and Methods

### Mice

Blood was drawn from the retroorbital plexus of gene-targeted mice lacking annexin A7 (*anx7^−/−^*) and corresponding 129 SV wild type mice (*anx7^+/+^*) into heparin-coated tubes. The erythrocytes were washed two times with Ringer solution containing 125 mM NaCl, 5 mM KCl, 1 mM MgSO_4_, 32 mM N-2-hydroxyethylpiperazine-N-2-ethanesulfonic acid (HEPES), 5 mM Glucose, 1 mM CaCl_2_, pH = 7.4. The generation and properties of *anx7^−/−^* mice were described earlier [8]. The work was carried out in accordance with the code of ethics for experiments involving animals as well as the German law for the welfare of animals and has been approved by the respective authority (Regierungspräsidium Tübingen).

### Solutions and Chemicals

Erythrocytes were incubated *in vitro* at a hematocrit of 0.4% in Ringer solution containing (in mM) 125 NaCl, 5 KCl, 1 MgSO_4_, 32 N-2-hydroxyethylpiperazine-N-2-ethanesulfonic acid (HEPES), 5 glucose, 1 CaCl_2_; pH 7.4 at 37°C for 48 hours. Where indicated, extracellular glucose was removed for 12 hours, 1 µM Ca^2+^ ionophore ionomycin (Sigma, Schnelldorf, Germany) applied for 30 min, hyperosmotic shock (addition of 550 mM sucrose for 2 hours) induced or 5 µl/ml annexin V or 4 µg/ml antibody directed against the chemokine ligand 16 (CXCL16) added to the respective solutions.

### FACS Analysis of Annexin V-binding and Forward Scatter

After incubation under the respective experimental conditions, 50 µl cell suspension were washed in Ringer solution containing 5 mM CaCl_2_ and then stained for 20 minutes with Annexin-V-Fluos (1∶500 dilution; Roche, Mannheim, Germany) under protection from light [43]. In the following, the forward scatter (FSC) of the cells was determined and annexin V fluorescence intensity was measured in FL-1 with an excitation wavelength of 488 nm and an emission wavelength of 530 nm on a FACS Calibur (BD, Heidelberg, Germany).

### Cell Culture of Human Umbilical Vein Endothelial Cells (HUVEC)

Human umbilical vein endothelial cells (HUVEC) from Promocell (Heidelberg, Germany), listed as CRL 1730 by the American Type Culture Collection database, were grown to confluency in complete endothelial cell basal medium from Lifeline cell-system (Kirkland, USA) containing growth factors and 10% fetal bovine serum from Lifeline Cell-Systems (Kirkland, USA). Erythrocyte adhesion to HUVEC cells was determined as described previously [37].

### Dynamic Erythrocyte Adhesion to Endothelium *in vitro*

Cultured HUVEC (5×10^5^) were attached on sterile coverslips coated with 0.2% gelatine (Sigma-Aldrich) by overnight incubation in complete endothelial cell basal medium under cell culture conditions for 24 hours. Erythrocytes prepared as indicated were perfused on a HUVEC monolayer in a flow chamber (Harvard, USA) at arterial shear rates (1200^−s^). The interaction events were recorded with a CCD camera (Carl Zeiss) with 20×magnification, followed by analysis of the number adherent erythrocytes per high powerfield.

### Erythrocyte Adhesion and Tissue Injury Following Acute Renal Failure

Annexin A7 knockout (*anx7^−/−^*) and wild type mice (*anx7^+/+^*) were kept on a regular 12 h dark-light cycle with free access to rodent chow and tap water. Animals were anesthetized and placed on a temperature-controlled heating table (RT, Effenberg, Munich, Germany) to maintain body temperature at 37.0°C for renal ischemia/reperfusion procedure as described [44]. Briefly, right flank incision was performed with help of a coagulation electrode (Erbe, ICC50, Tübingen, Germany) to prevent bleeding of muscle and skin vessels. The renal pedicle, including the renal artery and renal vein, were ligated using a 4/0 silk suture, and the right kidney was removed without interfering with the adrenal vessels. The surgical wound was closed using 5/0 nylon sutures. Animals were then placed in a right lateral decubitus position and a left flank incision was performed. Operations were performed under an upright dissecting microscope (Leica, MZ95, Bensheim, Germany). The left kidney was carefully removed from connective tissues, avoiding the adrenal gland and vessels and the kidney positioned in a lucite cup. The kidney was kept wet and warm with a wet swab soaked with water at 37.0°C. The vessel was dissected from adjacent tissues, close to its takeoff from the abdominal aorta, then an 8/0 nylon suture (Ethicon, Norderstedt, Germany) was placed around the artery. This technique allows for interruption of only the arterial blood flow to the kidney without compression of the renal vein. The suture was placed over a small pole and a weight of 1 g was attached to each end immediately followed by occlusion of the artery. Successful occlusion was confirmed by a change in color from red to white. After the experimental procedure (45 minutes ischemia), the left kidney was reperfused by removal of the hanging weights and returned into its retroperitoneal position. The surgical wound was closed using 5/0 nylon sutures. Following 24 hours after reperfusion mice were sacrificed and kidneys were perfused with PBS followed by perfusion with 4% paraformaldehyde.

### Morphologic Evaluation of Renal Histology

For histological analyses, 4% paraformaldehyde fixed kidneys were with, dehydrated in ethanol and xylol followed by embedding in paraffin. Paraffin-embedded tissues were cut into sections of 2 µm thickness and stained with periodic acid Schiff (PAS) and Sirius red stain. Renal morphology was investigated by light microscopy as described below with the investigator being blinded to the genotype of the mice. Tubulointerstitial damage, i.e. tubular necrosis, tubular atrophy and tubular dilation was assessed on PAS-stained paraffin sections at a magnification of x200 using a semiquantitative scoring system (acute tubular necrosis score, ATN). For determination of ATN, 15 fields per kidney were randomly sampled and graded as follows: grade 0, no change; grade 1, necrosis involving less than 25% of the area; grade 2, necrosis affecting 25–50%; grade 3, necrosis involving more than 50%, and grade 4 involving (almost) the entire area. Erythrocyte attachment was monitored in the inner stripe of diseased kidneys in 12 fields at 200× magnification and graded using a scoring system from 0 to 4. (Grade 0, no Erythrocytes attached; grade 1, sporadic erythrocytes attached; grade 2, attached erythrocytes in more than 25% of the capillaries; grade 3, attached erythrocytes in more than 50% of the capillaries; grade 4, attached erythrocytes in more than 75% of the capillaries.).

### Statistics

Data are expressed as arithmetic means ± SEM. Statistical analysis was made using *t* test or paired ANOVA with Tukey’s test as post-test, as indicated in the figure legends. n denotes the number of different erythrocyte specimens studied. The batches of erythrocytes differed moderately in their susceptibility to eryptosis. Thus, the control values were not identical in all series of experiments. To avoid any bias potentially introduced by the use of different erythrocyte batches, comparisons were always made within a given erythrocyte batch.

## Results

**Figure 1:**
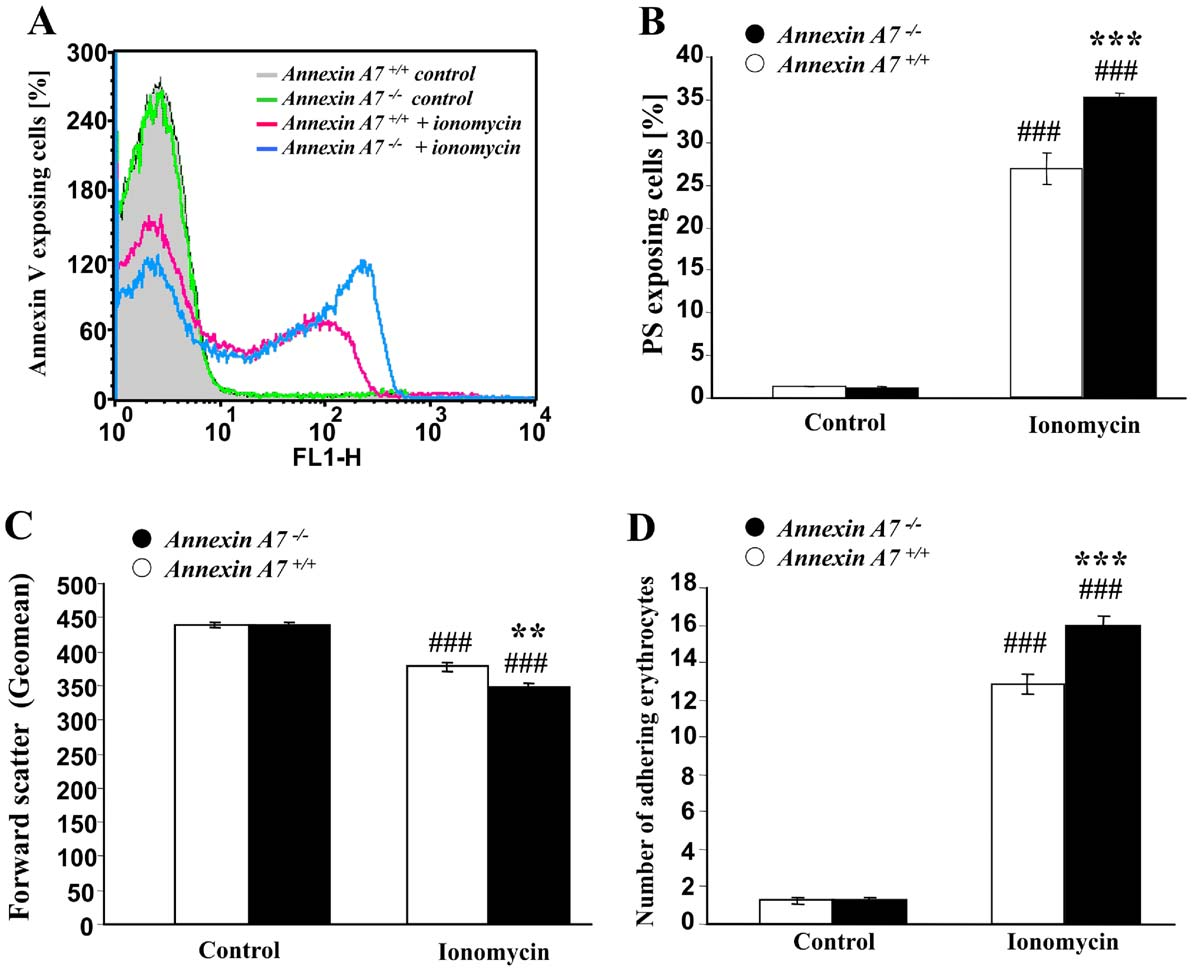
Enhanced ionomycin induced eryptosis and adhesion of erythrocytes from annexin7-deficient mice. A. Histogram of annexin V-binding reflecting phosphatidylserine exposure in a representative experiment of erythrocytes from annexin7-deficient mice (*anx7^−/−^*) and their wild type control mice (*anx7^+/+^*) exposed for 30 min to Ca^2+^ ionophore ionomycin (1 µM). **B.** Arithmetic means ± SEM (n = 8−9) of the percentage of annexin V-binding erythrocytes from annexin7-deficient mice (*anx7^−/−^*, black bars) and their wild type control mice (*anx7^+/+^*, white bars) exposed for 30 min to Ringer without (left bars) or with (right bars) ionomycin (1 µM). ### significant (p<0.001) difference from absence of ionomycin, *** significant difference (p<0.001) from *anx7^+/+^* erythrocytes (ANOVA). **C.** Arithmetic means ± SEM (n = 8−9) of the forward scatter of erythrocytes from annexin7-deficient mice (*anx7^−/−^*, black bars) and their wild type control mice (*anx7^+/+^*, white bars) exposed for 30 min to Ringer without (left bars) or with (right bars) ionomycin (1 µM). ### significant (p<0.001) difference from absence of ionomycin, *** significant difference (p<0.001) from *anx7^+/+^* erythrocytes (ANOVA). **D.** Arithmetic means ± SEM (n = 6) of the number of erythrocytes from annexin7-deficient mice (*anx7^−/−^*, black bars) and their wild type control mice (*anx7^+/+^*, white bars) adhering to the human umbilical vein endothelial cells (HUVEC) following exposure for 30 min to Ringer without (left bars) or with (right bars) ionomycin (1 µM). ### significant difference (p<0.001) from absence of ionomycin, *** significant difference (p<0.001) from *anx7^+/+^* erythrocytes (ANOVA).

Ionomycin was utilzed to increase cytosolic Ca^2+^ concentration in erythrocytes from gene-targeted mice lacking annexin A7 (*anx7^−/−^*) and from their corresponding wild type mice (*anx7^+/+^*). As shown in Fig. 1, the exposure of erythrocytes to ionomycin (1 µM) stimulated cell membrane scrambling thus increasing the percentage of phosphatidylserine exposing and annexin V binding erythrocytes. The effect was slightly but significantly more pronounced in *anx7^−/−^* than in *anx7^+/+^* erythrocytes (Fig. 1A,B). The increase of cytosolic Ca^2+^ concentration by ionomycin treatment further resulted in a significant decrease of erythrocyte forward scatter reflecting cell shrinkage (Fig. 1C). Again, the effect was slightly but significantly more pronounced in *anx7^−/−^* than in *anx7^+/+^* erythrocytes. As shown in Fig. 1D, treatment with ionomycin further enhanced the percentage of erythrocytes adhering to human umbilical vein endothelial cells (HUVEC), an effect again significantly more pronounced in *anx7^−/−^* than in *anx7^+/+^* erythrocytes.

**Figure 2:**
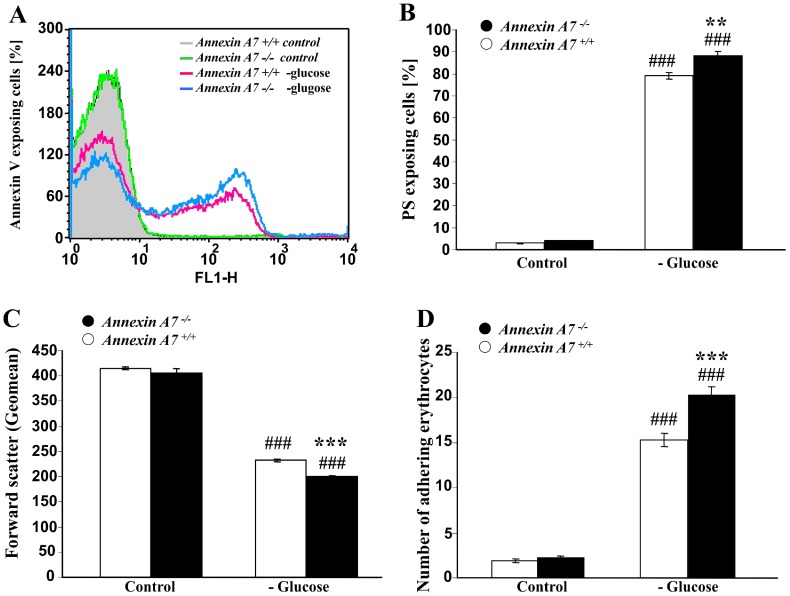
Enhanced eryptosis and adhesion of erythrocytes from annexin7-deficient mice following glucose depletion. A. Histogram of annexin V-binding reflecting phosphatidylserine exposure in a representative experiment of erythrocytes from annexin7-deficient mice (*anx7^−/−^*) and their wild type control mice (*anx7^+/+^*) exposed for 12 hours to glucose-depleted Ringer. **B.** Arithmetic means ± SEM (n = 8−9) of the percentage of annexin V-binding erythrocytes from annexin7-deficient mice (*anx7^−/−^*, black bars) and their wild type control mice (*anx7^+/+^*, white bars) exposed for 12 hours to glucose-containing (left bars) or glucose-depleted (right bars) Ringer. ### significant (p<0.001) difference from glucose-containing Ringer, *** significant difference (p<0.001) from *anx7^+/+^* erythrocytes (ANOVA). **C.** Arithmetic means ± SEM (n = 8−9) of the forward scatter of erythrocytes from annexin7-deficient mice (*anx7^−/−^*, black bars) and their wild type control mice (*anx7^+/+^*, white bars) exposed for 12 hours to glucose-containing (left bars) or glucose-depleted (right bars) Ringer. ### significant (p<0.001) difference from glucose-containing Ringer, *** significant difference (p<0.001) from *anx7^+/+^* erythrocytes (ANOVA). **D.** Arithmetic means ± SEM (n = 6) of the number of erythrocytes from annexin7-deficient mice (*anx7^−/−^*, black bars) and their wild type control mice (*anx7^+/+^*, white bars) adhering to human umbilical vein endothelial cells (HUVEC) following exposure of the erythrocytes for 12 hours to glucose-containing (left bars) or glucose-depleted (right bars) Ringer. ### significant (p<0.001) difference from glucose-containing Ringer, *** significant difference (p<0.001) from *anx7^+/+^* erythrocytes (ANOVA).

Annexin7 deficiency further sensitized erythrocytes to the eryptotic effects of energy depletion. As shown in Fig. 2, energy depletion by removal of glucose resulted within 12 hours in a significant increase of the percentage of annexin V-binding erythrocytes reflecting erythrocytes exposing phosphatidylserine at their surface (Fig. 2A,B). The effect was slightly, but significantly, more pronounced in *anx7^−/−^* than in *anx7^+/+^* erythrocytes. Glucose removal further decreased the forward scatter, reflecting erythrocyte shrinkage (Fig. 2C). Again, the effect was significantly more pronounced in *anx7^−/−^* than in *anx7^+/+^* erythrocytes. As shown in Fig. 2D, glucose depletion further enhanced the percentage of erythrocytes adhering to HUVEC, an effect again significantly more pronounced in *anx7^−/−^* than in *anx7^+/+^* erythrocytes.

**Figure 3:**
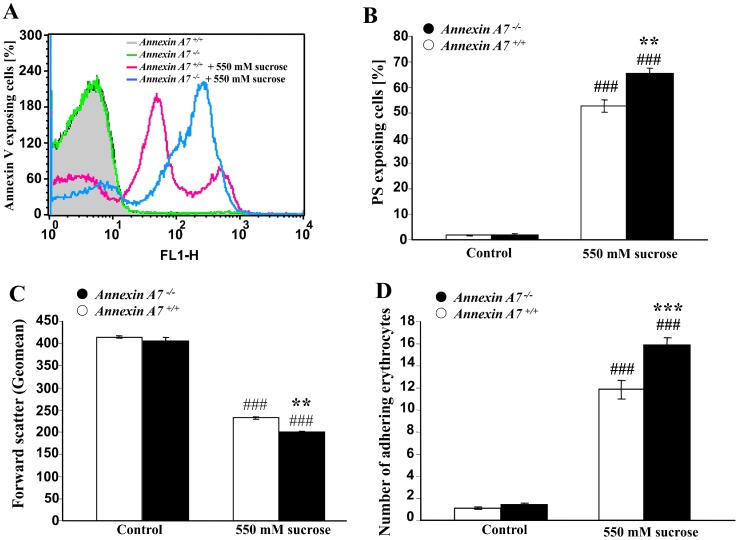
Enhanced eryptosis and adhesion of erythrocytes from annexin7-deficient mice following osmotic shock. A. Histogram of annexin V-binding reflecting phosphatidylserine exposure in a representative experiment of erythrocytes from annexin7-deficient mice (*anx7^−/−^*) and their wild type control mice (*anx7^+/+^*) exposed for 2 hours to hyperosmotic shock (550 mM sucrose added). **B.** Arithmetic means ± SEM (n = 8−9) of the percentage of annexin V-binding erythrocytes from annexin7-deficient mice (*anx7^−/−^*, black bars) and their wild type control mice (*anx7^+/+^*, white bars) exposed for 2 hours to isotonic (left bars) or hyperosmotic (550 mM sucrose added, right bars) Ringer. ### significant (p<0.001) difference from isotonic Ringer, *** significant difference (p<0.001) from *anx7^+/+^* erythrocytes (ANOVA). **C.** Arithmetic means ± SEM (n = 8−9) of the forward scatter of erythrocytes from annexin7-deficient mice (*anx7^−/−^*, black bars) and their wild type control mice (*anx7^+/+^*, white bars) exposed for 2 hours to isotonic (left bars) or hyperosmotic (550 mM sucrose added, right bars) Ringer. ### significant (p<0.001) difference from isotonic Ringer, *** significant difference (p<0.001) from *anx7^+/+^* erythrocytes (ANOVA). **D.** Arithmetic means ± SEM (n = 6) of the number of erythrocytes from annexin7-deficient mice (*anx7^−/−^*, black bars) and their wild type control mice (*anx7^+/+^*, white bars) adhering to human umbilical vein endothelial cells (HUVEC) following exposure of the erythrocytes for 2 hours to isotonic (left bars) or hyperosmotic (550 mM sucrose added, right bars) Ringer. ### significant (p<0.001) difference from isotonic Ringer, *** significant difference (p<0.001) from *anx7^+/+^* erythrocytes (ANOVA).

Similar observations were made under hyperosmotic shock, which was induced by addition of 550 mM sucrose to isotonic Ringer solution. As shown in Fig. 3, hyperosmotic shock was within 2 hours followed by a significant increase of the percentage of annexin V-binding erythrocytes (Fig. 3A,B), an effect significantly more pronounced in *anx7^−/−^* than in *anx7^+/+^* erythrocytes. Hyperosmotic shock further decreased forward scatter (Fig. 2C), an effect again significantly more pronounced in *anx7^−/−^* than in *anx7^+/+^* erythrocytes. Osmotic shock further enhanced the percentage of erythrocytes adhering to HUVEC, an effect again significantly more pronounced in *anx7^−/−^* than in *anx7^+/+^* erythrocytes (Fig. 3D).

**Figure 4:**
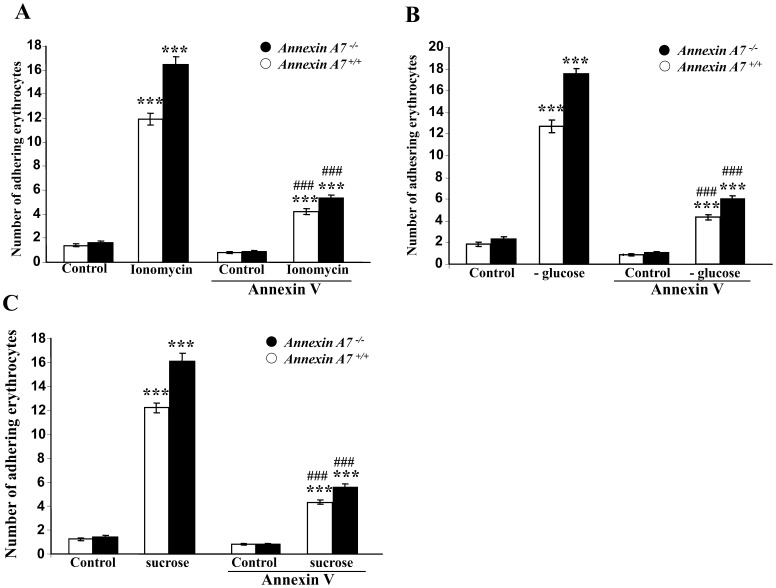
Role of phosphatidylserine exposure in dynamic adhesion of annexin-treated erythrocytes to endothelial cells under arterial shear stress. A. Arithmetic means ± SEM (n = 6) of erythrocytes from annexin7-deficient mice (*anx7^−/−^*, black bars) and their wild type control mice (*anx7^+/+^*, white bars) binding to human umbilical vein endothelial cells (HUVEC) under flow following exposure for 30 minutes to Ringer solution without (control) or with 1 µM ionomycin without or with a prior 30 min treatment with Annexin V (5 µl/ml). ***(p<0.001) indicates statistically significant difference from absence of ionomycin, ###(p<0.001) indicates statistically significant difference from absence of annexin V (ANOVA). **B.** Arithmetic means ± SEM (n = 6) of erythrocytes from annexin7-deficient mice (*anx7^−/−^*, black bars) and their wild type control mice (*anx7^+/+^*, white bars) binding to human umbilical vein endothelial cells (HUVEC) under flow following a 12 hours treatment with Ringer solution with (control), or without (-Glucose) glucose without or with a prior 30 min treatment with Annexin V (5 µl/ml). ***(p<0.001) indicates statistically significant difference from absence of glucose, ###(p<0.001) indicates statistically significant difference from absence of annexin V (ANOVA). C. Arithmetic means ± SEM (n = 6) of erythrocytes from annexin7-deficient mice (*anx7^−/−^*, black bars) and their wild type control mice (*anx7^+/+^*, white bars) binding to human umbilical vein endothelial cells (HUVEC) under flow following a 2 hours exposure to isotonic (control) or hypertonic (550 mM sucrose) Ringer solution without or with a prior 30 min treatment with Annexin V (5 µl/ml). ***(p<0.001) indicates statistically significant difference from absence of hyperosmotic shock, ###(p<0.001) indicates statistically significant difference from absence of annexin V (ANOVA).

Additional experiments were performed to investigate whether enhanced vascular adhesion of Annexin A7 erythrocytes following ionomycin-treatment requires phosphatidylserine exposure at the cell surface. To this end, phosphatidylserine on the erythrocyte surface was blocked with annexin-V, which firmly binds to and thus masks phosphatidylserine. As illustrated in (Fig. 4A), the increased adhesion of ionomycin-treated erythrocytes to HUVEC under flow at shear rates of 1200^−s^ was significantly attenuated in the presence of annexin-V (5 µl/ml). Similar observations were made following exposure of the erythrocytes to energy depletion (Fig. 4B) and following exposure of the erythrocytes to hyperosmotic shock (Fig. 4C).

**Figure 5:**
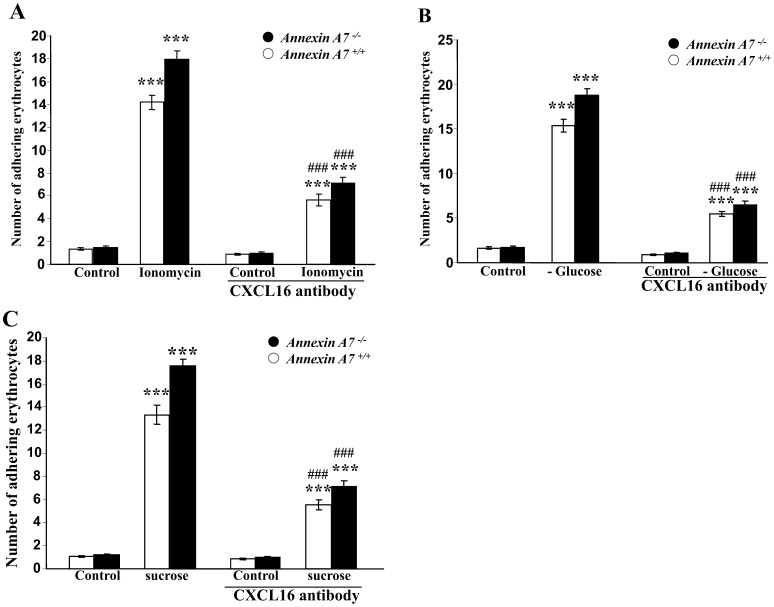
Role of CXCL16 in dynamic adhesion erythrocytes to anti-CXCl16-treated endothelial cells under arterial shear stress. A. Arithmetic means ± SEM (n = 6) of erythrocytes from annexin7-deficient mice (*anx7^−/−^*, black bars) and their wild type control mice (*anx7^+/+^*, white bars) binding to human umbilical vein endothelial cells (HUVEC) under flow. The erythorcytes were pretreated for 30 minutes with Ringer solution without (control) or with 1 µM ionomycin. The HUVEC were left untreated or treated for 2 hours with neutralizing antibody directed against endothelial CXCL16 (4 µg/ml), ***(p<0.001) indicates statistically significant difference from absence of ionomycin (1 µM), ###(p<0.001) indicates statistically significant difference from anti CXCL 16 (ANOVA). **B.** Arithmetic means ± SEM (n = 6) of erythrocytes from annexin7-deficient mice (*anx7^−/−^*, black bars) and their wild type control mice (*anx7^+/+^*, white bars) binding to human umbilical vein endothelial cells (HUVEC) under flow. The erythrocytes were pretreated for 12 hours with Ringer with (control), or without (-Glucose) glucose. The HUVEC were left untreated or treated for 2 hours with neutralizing antibody directed against endothelial CXCL16 (4 µg/ml), ***(p<0.001) indicates statistically significant difference from absence of glucose, ###(p<0.001) indicates statistically significant difference from anti CXCL 16 (ANOVA). C. Arithmetic means ± SEM (n = 6) of erythrocytes from annexin7-deficient mice (*anx7^−/−^*, black bars) and their wild type control mice (*anx7^+/+^*, white bars) binding to human umbilical vein endothelial cells (HUVEC) under flow. The erythrocytes were pretreated for 2 hours with isotonic (control) or hypertonic (550 mM sucrose) Ringer. The HUVEC were left untreated or treated for 2 hours with neutralizing antibody directed against endothelial CXCL16 (4 µg/ml), ***(p<0.001) indicates statistically significant difference from absence of hyperosmotic shock, ###(p<0.001) indicates statistically significant difference from anti CXCL 16 (ANOVA).

Since CXCL16 has previously been shown to bind phosphatidylserine and to be thus involved in cell adhesion [37], [45], [46], further experiments were performed to test whether the binding of ionomycin-treated erythrocytes to endothelium under flow conditions involves CXCL16. Therefore, HUVEC were exposed to an antibody directed against CXCL16 (4 µg/ml). As shown in Fig. 5A, the endothelial adhesion of ionomyin-treated erythrocytes was significantly less pronounced following exposure of HUVEC cells to CXCL16-blocking antibody than following treatment of HUVEC with isotype control antibody of the same concentration. Again, similar observations were made following exposure of the erythrocytes to energy depletion (Fig. 5B) and following exposure of the erythrocytes to hyperosmotic shock (Fig. 5C).

**Figure 6:**
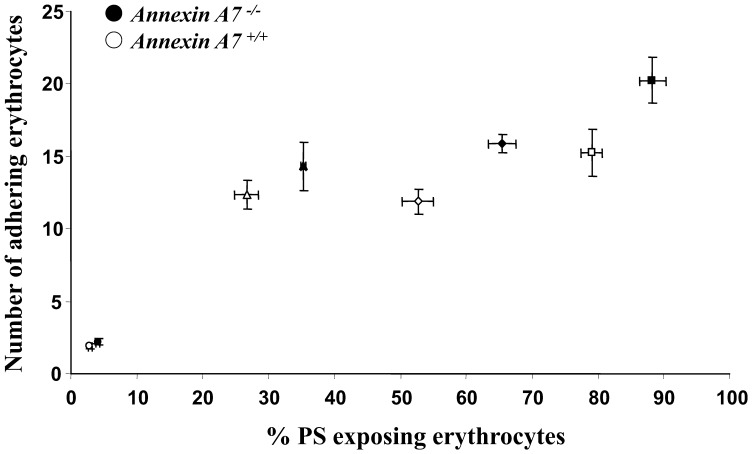
Correlation between phosphatidylserine exposure and dynamic adhesion of erythrocytes to endothelial cells under arterial shear stress. Arithmetic means ± SEM (n = 6−8) of the erythrocytes adhering to HUVEC as a function of the percentage erythrocytes binding annexin V. The erythrocytes from annexin7-deficient mice (*anx7^−/−^*, closed symbols) and their wild type control mice (*anx7^+/+^*, open symbols) were left without pretreatment (circles), or pretreated 30 minutes with 1 µM ionomycin (triangles), 12 hours with glucose-depleted Ringer (squares) or 2 hours with hyperosmotic shock by addition of 550 mM sucrose (diamonds).

Plotting of erythrocyte adhesion to HUVEC against the percentage of phosphatidylserine exposing erythrocytes (Fig. 6) revealed that at any given phosphatidylserine exposure the adhesion of *anx7^−/−^* and *anx7^+/+^* erythrocytes was similar. Thus, increased adhesion of annexin7 deficient erythrocytes was fully explained by their enhanced susceptibility towards triggers of cell membrane scrambling.

**Figure 7:**
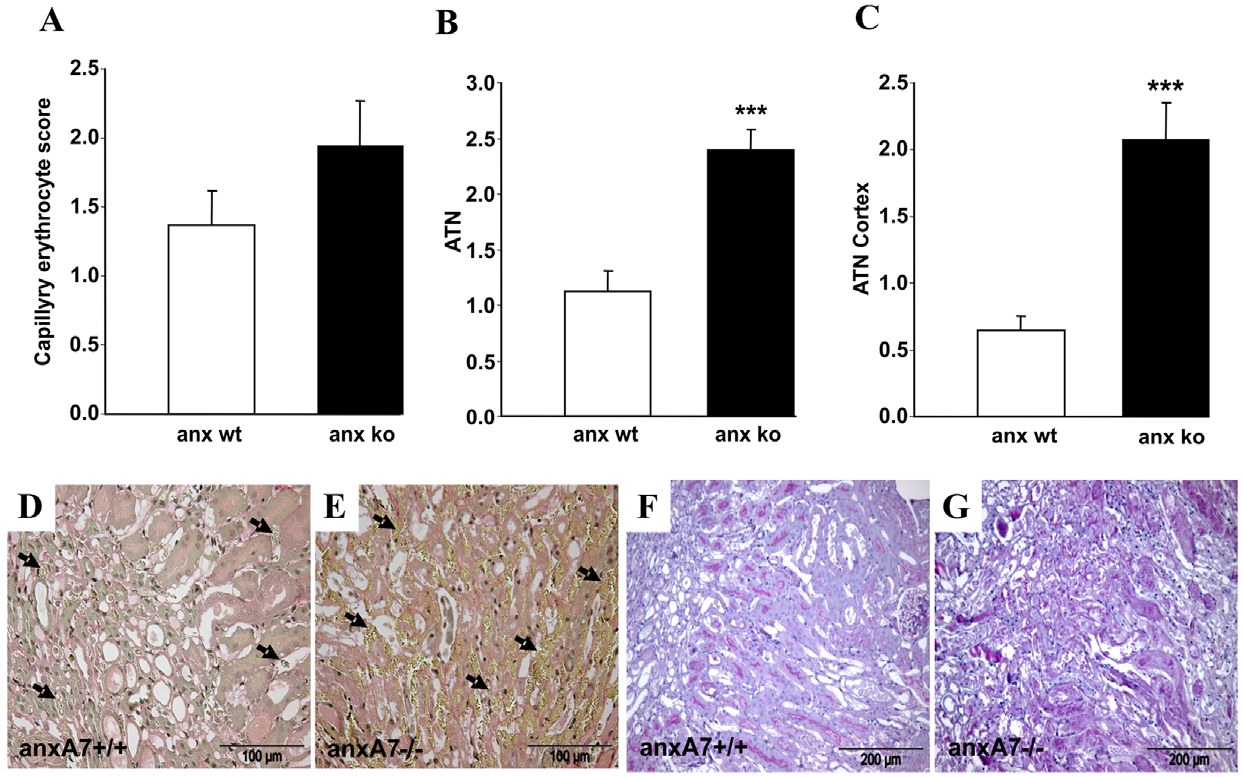
Annexin-binding erythrocytes in ischemic kidneys from wild type and from annexin deficient mice. A. Arithmetic means ± SEM (n = 7) of erythrocyte abundance in the inner stripe of renal tissue following renal ischemia/reperfusion of anx7^−/−^ mice (black bar) and *anx7^+/+^* mice (white bar). **B.** Arithmetic means ± SEM (n = 7) of the acute tubular necrosis (ATN) score in the inner stripe of renal tissue following renal ischemia of anx7^−/−^ mice and *anx7^+/+^* mice, ***(p<0.001) indicate significant difference to wild type mice (ANOVA). **C.** Arithmetic means ± SEM (n = 7) of the acute tubular necrosis (ATN) score in the outer cortex of renal tissue following renal ischemia of anx7^−/−^ mice and *anx7^+/+^* mice, ***(p<0.001) indicate significant difference to wild type mice (ANOVA). **D, E.** Representative light microscopy image from kidney section of ischemic *anx7^+/+^* mice (**D**) and anx7^−/−^ mice (**E**) stained with sirius red. Arrows indicate yellow stained erythrocytes within renal capillaries. **F, G.** Representative light microscopy image from kidney section of ischemic *anx7^+/+^* mice (**F**) and anx7^−/−^ mice (**G**) stained with PAS.

To test for a potential consequence of erythrocyte adhesion for microcirculation *in vivo*, kidneys were subjected to ischemia (45 min) and subsequently analysed by light microscopy. 24 hours after induction of ischemia/reperfusion model abundance of erythrocytes tended to be increased in *anx7^−/−^* in ischemic kidneys at the inner stripe (Fig. 7A; E arrows) compared to *anx7^+/+^* mice (Fig. 7A; D arrows). This effect did not reach statistical significance due to high variability. However, kidneys from *anx7^+/+^* mice showed only mild tubular necrosis with partial loss of tubular brush border (Fig. 7F). In contrast, ischemia/reperfision in *anx7^−/−^* mice resulted in significantly enhanced tubular necrosis with cast formation and more pronounced loss of the tubular brush border (Fig. 7B; C; G).

## Discussion

The present study confirms previous observations revealing enhanced susceptibility of annexin7 deficient erythrocytes to stimulators of eryptosis [10], [11]. More importantly, the present observations reveal that the enhanced eryptosis of annexin7 deficient erythrocytes is paralleled by increased adhesion of affected erythrocytes to endothelial cells.

As shown earlier [10], PGE_2_-formation, cation currents, increase of cytosolic Ca^2+^ concentration ([Ca^2+^]_i_), and cell membrane scrambling were all more pronounced in *anx7^−/−^* than in *anx7^+/+^*-erythrocytes. The difference was blunted following inhibition of cyclooxygenase by aspirin or diclofenac [10]. Increase of cytosolic Ca^2+^ concentration is well known to trigger erythrocyte cell membrane scrambling with subsequent phosphatidylserine exposure at the cell surface [26], [28]. Cytosolic Ca^2+^ is further known to activate Ca^2+^ sensitive K^+^ channels [23], [24] resulting in cell shrinkage due to exit of K^+^, hyperpolarisation of the cell membrane, exit of Cl^−^ and thus cellular loss of KCl with osmotically obliged water [25].

Coating of phosphatidylserine at the erythrocyte surface by annexin V interfered with the engulfment of the eryptotic cells by macrophages [11] and interfered with binding of phosphatidylserine exposing erythrocytes to human endothelial cells (HUVEC) [37].

Adhesion of phosphatidylserine exposing cells to the endothelial cells presumably impairs microcirculation [38]–[42]. The derangement of microcirculation presumably contributes to the pathophysiology of clinical disorders associated with enhanced eryptosis [12], including iron deficiency [47], phosphate depletion [48], Hemolytic Uremic Syndrome [49], sepsis [50], sickle cell disease [11], malaria [11], [51]–[54], Wilson’s disease [55] and presumably metabolic syndrome [56]. Adhesion of eryptotic cells may further complicate the effects of xenobiotics and other small molecules known to trigger eryptosis [36], [56]–[86].

At least in theory, adhesion of eryptotic erythrocyte in renal medulla could contribute to acute ischemic renal failure. The high Cl^−^ and urea concentrations prevailing in renal medulla counteract erythrocyte phosphatidylserine exposure [87] and at normal renal blood flow the contact time of erythocytes within the renal medulla is too short to trigger significant phosphatidylserine exposure. The contact time is, however, markedly increased in acute renal failure which is typically paralleled by trapping of erythrocytes in the transition from medulla to cortex [88]. Following ischemia, the abundance of erythrocytes tended to be higher in *anx7^−/−^* mice and *anx7^+/+^* mice., a difference, however, not reaching statistical significance. Ischemia was followed by tubular necrosis, which was significantly more pronounced in *anx7^−/−^* mice than in *anx7^+/+^* mice. It remains uncertain, however, whether this difference on renal injury is due to impact of erythrocytes on microcirculation, due to altered function of other blood cells or due to differences in renal epithelial cells. Following ischemia/reperfusion renal cells may undergo apoptosis, programmed necrosis or necroptosis [89]–[95]. Mechanisms involved in or resulting from cell injury following acute renal ischemia include excessive production of nitric oxide and free radicals, tyrosine nitrosylation, caspase-8, receptor-interacting protein kinase 3 (RIP3), mitochondrial permeability transition, Phospholipase A(2), PGE_2_, poly(ADP-ribose) polymerase, calpain, malondialdehyde, superoxide dismutase, lysophospholipids and free fatty acids [89], [90], [92], [94]–[96]. Possibly, annexin7 deficiency sensitizes not only erythrocytes but as well other cell types, such as renal epithelia, to the effects of energy depletion and thus enhances, for instance PGE_2_ formation in epitheilal cells. Clearly, further experimental effort is needed to define the mechanisms accounting for the enhanced vulnerability of renal tissues in *anx7^−/−^* mice following ischemia.

In conclusion, the present observations show that the enhanced susceptibility of annexin7 deficient erythrocytes is paralleled by enhanced adhesion to human umbilical vein endothelial cells. The enhanced adhesion of eryptotic erythrocytes is expected to compromize microcirculation and thus to participate in the pathophysiology of disorders with excessive eryptosis.
